# Tissue-Specific Role of Macrophages in Noninfectious Inflammatory Disorders

**DOI:** 10.3390/biomedicines8100400

**Published:** 2020-10-09

**Authors:** Daria Skuratovskaia, Maria Vulf, Olga Khaziakhmatova, Vladimir Malashchenko, Aleksandra Komar, Egor Shunkin, Valeriya Shupletsova, Andrei Goncharov, Olga Urazova, Larisa Litvinova

**Affiliations:** 1 Center for Immunology and Cellular Biotechnology, Immanuel Kant Baltic Federal University, 236001 Kaliningrad, Russia; mary-jean@yandex.ru (M.V.); olga_khaziakhmatova@mail.ru (O.K.); VlMalashchenko@kantiana.ru (V.M.); alexandkomar@gmail.com (A.K.); egor.shunkin@gmail.com (E.S.); vshupletsova@mail.ru (V.S.); agoncharov59@mail.ru (A.G.); larisalitvinova@yandex.ru (L.L.); 2 Pathophysiology Division, Siberian State Medical University, 634050 Tomsk, Russia; urazova72@yandex.ru

**Keywords:** obesity, inflammation, macrophage, adipose tissue, liver, NAFLD, endothelial dysfunction, atherosclerosis, nervous tissue

## Abstract

Chronic inflammation may not begin with local tissue disorders, such as hypoxia, but with the accumulation of critically activated macrophages in one site. The purpose of this review is to analyze the data reported in the scientific literature on the features of the functions of macrophages and their contributions to the development of pathology in various tissues during aseptic inflammation in obese subjects. In individuals with obesity, increased migration of monocytes from the peripheral blood to various tissues, the proliferation of resident macrophages and a change in the balance between alternatively activated anti-inflammatory macrophages (M2) and pro-inflammatory classically activated macrophages (M1) towards the latter have been observed. The primary cause of some metabolic pathologies has been precisely identified as the recruitment of macrophages with an altered phenotype, which is probably typical for many other pathologies. Recent studies have identified phenotypes, such as metabolically activated M (MMe), oxidized (Mox), hemoglobin-related macrophages (Mhem and MHb), M4 and neuroimmunological macrophages (NAM, SAM), which directly and indirectly affect energy metabolism. The high heterogeneity of macrophages in tissues contributes to the involvement of these cells in the development of a wide range of immune responses, including pathological ones. The replenishment of tissue-specific macrophages occurs at the expense of infiltrating monocyte-derived macrophages (MoMFs) in the pathological process. The origin of MoMFs from a general precursor retains their common regulatory mechanisms and similar sensitivity to regulatory stimuli. This makes it possible to find universal approaches to the effect on these cells and, as a consequence, universal approaches for the treatment of various pathological conditions.

## 1. Introduction

An important aim of modern medicine is to develop new solutions for the treatment of socially important diseases. Obesity and its associated complications (e.g., type 2 diabetes mellitus (T2DM), diabetic foot, atherosclerosis, non-alcoholic fatty liver disease (NAFLD) and steatohepatitis) occupy a leading position among the causes of mortality in the global population [1]. Over four million people die each year due to being overweight or obese. Thus, the problem of obesity has grown to epidemic proportions. These diseases are multifactorial, with insufficiently defined initial pathogenic factors. However, based on the opinions of the international community and the studies conducted by our team (over 10 years), in most cases, metabolic complications are associated with inflammation [2,3,4,5,6,7,8]. Therefore, chronic subclinical inflammation contributes to the disruption of lipid and carbohydrate metabolism, and its local foci lead to dysfunction of various tissues (adipose tissue, liver, vascular endothelium, etc.). Several approaches may provide successful control. 

**Figure 1:**
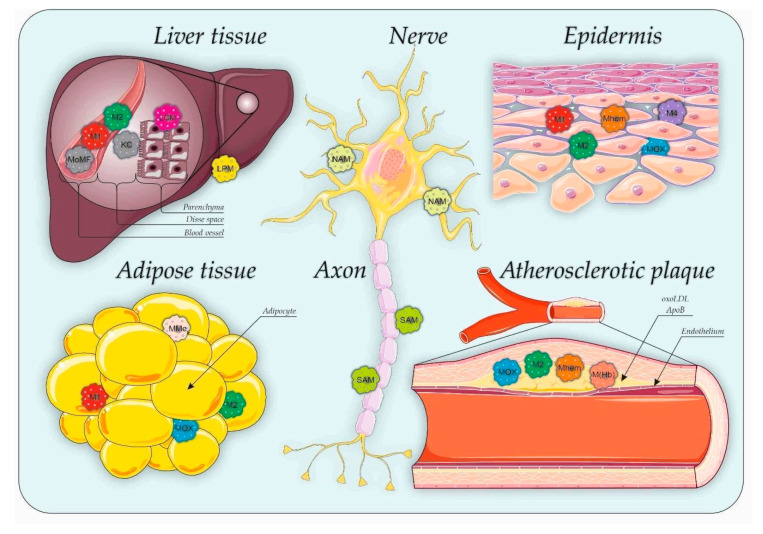
Tissue distribution of macrophages in non-infectious metabolic disorders. M1—inflammatory classically activated macrophages; M2—alternatively activated anti-inflammatory macrophages; M4—M4 macrophage; Mhem—hemoglobin-related macrophages; KC—Kupffer cells; LCM—liver capsular macrophages; LPM—large peritoneal macrophages; MoMF—monocyte-derived macrophages; Mox—oxidized macrophages; NAM—neuro-associated macrophages; SAM—sympathetic neuron-associated macrophages; MMe—metabolically activated macrophages; oxoLDL—oxidized low-density lipoprotein; ApoB—Apolipoprotein B. This figure has been created by modifying the templates from Servier Medical Art (https://smart.servier.com).

Macrophages are one of the key cell types responsible for maintaining their homeostasis and regulating inflammatory and regenerative processes. They are present in all organs and tissues (Figure 1), their circulating form is called a monocyte and the resident form is called a macrophage.

Macrophages are an extremely heterogeneous group of cells with a highly variable receptor profile and functional activity due to their plasticity. In this regard, several classifications have been reported in the scientific literature.

On the one hand, macrophages are classified according to their functional activity and stimuli that induce differentiation. According to this classification, a continuous spectrum of macrophage cell types is distinguished, the extreme categories of which are classically and alternatively activated macrophages (M1 and M2) [9]. Classical activation is due to interferons and proteins of the tumor necrosis factor (TNF) family (CD40 L), as well as some Toll-like receptor (TLR) ligands, such as lipopolysaccharide (LPS), and these cells are characterized by high pro-inflammatory activity and increased production of pro-inflammatory cytokines (TNF-α, Interleukin-1 (IL-1), etc.), inducible nitric oxide synthase (iNOS) and reactive oxygen species (ROS) [9,10]. Alternative activation occurs after stimulation with glucocorticoids and anti-inflammatory cytokines (IL-4 and IL-13) [11], and these cells are characterized by increased production of anti-inflammatory cytokines, such as TRF-β and IL-10, and they promote regenerative processes and the formation of the extracellular matrix [12].

On the other hand, several variants of the immunophenotypic classification of macrophages have been described. The classification based on differences in the level of expression of CD14 and CD16 molecules on the cell surface is the closest scheme to a functional classification. Within the framework of this classification, three large groups are distinguished: classical (CD14highCD16−), intermediate (CD14highCD16low) and non-classical macrophages [13,14,15]. Occasionally, non-classical and transitional macrophages are grouped together (CD14lowCD16low) [13]. Within the framework of this classification, classical macrophages (CD14highCD16−) exhibit pronounced phagocytic activity [16], while nonclassical macrophages are characterized by pro-inflammatory properties [14]. In addition, subpopulations of macrophages with differences in FcγR1 (CD64) expression have been distinguished. These macrophages are CD64+CD16+ cells that combine both the properties of macrophages and dendritic cells (DCs) and CD64-CD16+ cells that express the major histocompatibility complex II (MHC II) molecule at high levels and display a pronounced antigen-presenting function [17,18].

**Figure 2:**
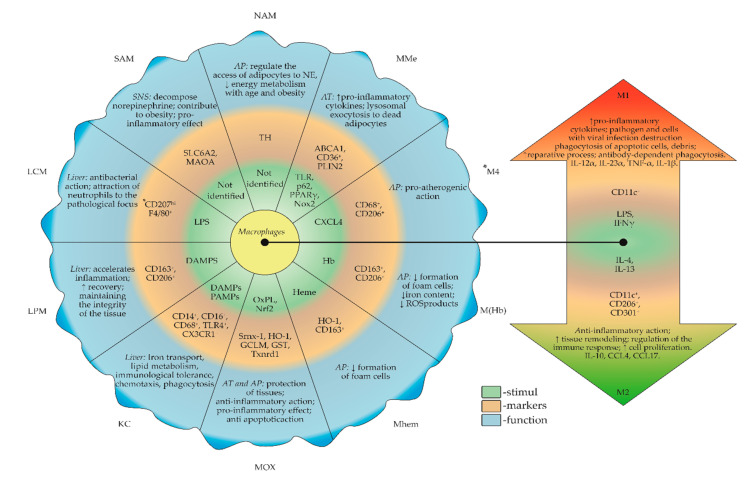
Inductors, markers and functions of macrophages subtypes in tissues. All macrophage subtypes may take a pro- or anti-inflammatory phenotype in obesity. MoMFs are a common precursor for subtypes of migrating macrophages in tissue. When damaged, MoMFs can assume a tissue-specific phenotype. Determining the exact origin of macrophages (resident or migratory) is a task in modern science. The plasticity of macrophages between M1 and M2 is also present in the resident forms and is shown by an arrow. The arrow indicates the stimuli, markers and functions that characterize both types of polarization. ABCA1—ATP binding cassette subfamily a member 1; AP—atherosclerosis plaque; AT—adipose tissue; DAMPS—damage-associated molecular patterns; GCLM—glutamate–cysteine ligase modifier subunit; GST—glutathione S-transferase; Hb/Hp—hemoglobin/haptoglobin; HO-1—heme oxygenase 1; IL—Interleukin; KC—Kupffer cells; LCM—liver capsular macrophages; LPM— large peritoneal macrophages; LPS—lipopolysaccharide; MAOA—monoamine oxidase A; MoMF—monocyte-derived macrophage; Mox—oxidized macrophages; MR—mannose receptor; NAM—neuro-associated macrophages; NE—norepinephrine; Nox2—NADPH oxidase-2; OxPL—oxidized phospholipids; PLIN2—Perilipin 2; PPARγ—peroxisome proliferator-activated receptor gamma; ROS—reactive oxygen species; SAM—sympathetic neuron-associated macrophages; SLC6A2—carrier family 6 member 2; SNS—sympathetic nervous system; Srnx-1—sulfiredoxin-1; TH—tyrosine hydroxylase; TLR—Toll-like receptor; Txnrd1—thioredoxin reductase 1; MMe—metabolically activated macrophages; *—mouse-specific markers; #—cannot be M1 and M2. This figure has been created by modifying the templates from Servier Medical Art (https://smart.servier.com).

Currently, a new model of the origin and development of tissue macrophages has been proposed and substantial experimental confirmation has been provided. The concept of a “layered myeloid system” suggests that a population of resident macrophages that develops from progenitor cells in the yolk sac of embryos [19,20] and is formed from hematopoietic stem cells independently. Another macrophage population is passenger or transitory myeloid cells, which are formed from hematopoietic bone marrow stem cells [21]. Monocyte-derived macrophages (MoMFs) migrate from the bone marrow into the bloodstream. Different phenotypes in the blood MoMF subpopulation are associated with activation and maturation. MoMFs are recruited to the inflammation site and acquire characteristic, tissue-specific properties when stimulated with chemokines and other molecules involved in intercellular communication (Figure 2).

Resident or tissue-specific macrophages are more specialized cells. Most resident cells are macrophages that developed in parallel with the development of the tissue. The main function of resident macrophages is to maintain tissue homeostasis and eliminate old and apoptotic cells [22]. Resident macrophages have significant differences in receptor expression and functions, depending on their tissue localization (Kupffer cells, alveolar macrophages, osteoclasts, etc.). If necessary, the pool of these cells is replenished due to the spontaneous migration of cells from the bloodstream. The concept does not exclude the possibility that the population of tissue macrophages is supplemented by macrophages of “classical monocytic” origin. It is complicated to trace the origin of cells, despite the many evaluation methods, but they are not accurate enough. Since the origin, migration and differentiation of macrophages are different, their functions may differ substantially, providing new opportunities for further research [23,24].

The high heterogeneity of macrophages in tissues contributes to the involvement of these cells in the development of a wide range of immune responses, including pathological ones. The replenishment of tissue-specific macrophages occurs at the expense of MoMFs in the pathological process. The origin of MoMFs from a general precursor retains their common regulatory mechanisms and similar sensitivity to regulatory stimuli. This makes it possible to find universal approaches to the effect on these cells and, as a consequence, universal approaches for the treatment of various pathological conditions.

The present review aimed to analyze data presented in the scientific literature on the features of the functions of macrophages and their contributions to the development of pathology in various tissues during aseptic inflammation in individuals with obesity.

## 2. Role of Macrophages in the Pathogenesis of Obesity 

The pathogenesis of obesity has been studied for many years, but the urgency of the problem persists. In individuals with obesity, adipocyte growth increases hypertrophically, contributing to the development of hypoxia in adipose tissue (AT) [25,26]. Thus, one of the key factors underlying insulin resistance (IR), along with the inflammation of AT, may be the dysregulation of energy homeostasis in insulin-dependent tissues (AT and skeletal muscles). In individuals with obesity (given its systemic nature), the importance of AT in the mechanisms regulating metabolic processes increases substantially. A study of the metabolism of AT revealed the ability of adipocytes to produce a large number of adipokines and other mediators that affect the states of metabolic and immune processes. A better understanding of the processes of inflammation and endocrine regulation of AT would provide opportunities to identify new factors influencing the prevention and treatment of obesity and its complications. As a result, studies aiming to clarify the roles of chemokines and pro- and anti-inflammatory cytokines in the development of insulin resistance (IR) will facilitate the identification of molecular targets for effective, individually selected therapy.

## 3. Molecular Basis of Adipose Tissue Inflammation in Individuals with Obesity

Immune cells and macrophages are recruited to AT through inflammatory mediators (Figure 1) [25]. Necrotic adipocytes are phagocytosed by macrophages, which produce chemokines and pro-inflammatory mediators. This process forms an inflammatory cycle with positive feedback [25,26]. In AT, the expression of markers of endoplasmic reticulum stress is induced, which promotes protein degradation and the initiation of apoptosis to aggravate oxidative stress in cells [27]. The transcription factors involved in pro-inflammatory pathways, such as NF-κB, Signal transducer and activator of transcription (STAT) and Activator protein 1 (AP-1), activate subsequent signaling cascades and induce the expression of proteins that inhibit the insulin signaling pathway, which contributes to the development of IR [26,28].

The number of macrophages in AT from humans with obesity increases from 4% to 12% compared to healthy donors [29]. Chemokine expression is induced in hypertrophic adipocytes, which leads to the accumulation and activation of macrophages in AT [30]. Greater numbers of macrophages infiltrate visceral AT than subcutaneous tissue [31]. The increase in the number of macrophages in AT results from their recruitment by the chemokine receptor pathways CCR2/CCL2 and CCR1/CCL5 [32]. Macrophages promote adipocyte hypertrophy, and hypertrophic adipocytes produce chemokines and their receptors, which recruit monocytes/macrophages to AT.

Researchers have proposed that monocytes transition from one type of activated state to another, differentiate into macrophages and then the differentiation pathways diverge to M1- and M2-like types of mature macrophages [33,34]. However, monocyte plasticity has been observed, which is known as the so-called classical pro-inflammatory type. These cells first polarize into anti-inflammatory monocytes and then terminally differentiate into type II macrophages [33,35].

Thus, AT has been considered an organ of the immune system that regulates metabolic homeostasis. In particular, chronic subclinical inflammation in individuals with obesity contributes to the formation of IR due to the recruitment of immune cells to the AT.

## 4. Specialization of Macrophages in Adipose Tissue: MMe and Mox Macrophages

Traditionally, two phenotypes of macrophages have been identified: M1 and M2 [36]. This division of macrophages into phenotypes is convenient for understanding their heterogeneity. However, this classification does not adequately describe the function of macrophages in individuals with obesity. The main inducers of the phenotype reversal (M2 to M1) of macrophages in individuals with obesity include the following metabolic stimuli: high levels of glucose and insulin, free fatty acids (FFAs), oxidized phospholipids (OxPLs), low-density lipoproteins and end products of deep glycation. In turn, cytokines and OxPLs, which are produced by M1 and M2 macrophages, promote the development of macrophages into a population of metabolically activated macrophages (MMe) and oxidized macrophages (Mox) (Figure 1). MMe and Mox are also associated with IR. Mixed macrophage phenotypes have been observed in several experimental studies using laboratory animals and in obese patients [37,38].

Molecules such as ATP binding cassette subfamily a member 1 (ABCA1), CD36, and Perilipin 2 (PLIN2) are markers of the MMe cell surface (Figure 2). The main functions of MMe in the early and late stages of diet-induced obesity are to produce pro-inflammatory cytokines (a detrimental function) and promote lysosomal exocytosis of dead adipocytes (a beneficial function that promotes dead adipocyte clearance through lysosomal exocytosis) [39] (Figure 2). At the molecular level, at least two mechanisms underlying the development of macrophages into the MMe phenotype have been identified. The first pathway involves palmitate and the cell surface TLR, which controls the production of pro-inflammatory cytokines [40]. The second pathway involves the activation of p62 and peroxisome proliferator-activated receptor gamma (PPARγ) by palmitate internalization, which stimulates lipid metabolism and restricts inflammation [40]. The balance between these two processes determines the general response of macrophages to metabolic dysfunction (pro- or anti-inflammatory effect) and may produce complex phenotypes of macrophages, covering the spectrum between “M1-like” and “M2-like” states [40]. Consequently, MMe both aggravate and reduce the process of inflammation in the tissue.

MMe play a key role in the pathogenesis of obesity and insulin resistance due to their ability to utilize dead adipocytes. In studies by Coats B.R. et al. (2017), the ablation of NADPH oxidase-2 (Nox2), a key regulator of inflammatory cytokine expression and lysosomal exocytosis in MMe, in mice after a 16-week intervention with a high-fat diet (HFD) leads to the accumulation of large numbers of dead adipocytes and a lower level of inflammation than in wild-type mice [41]. Eventually, this process leads to severe IR, hepatosteatosis and visceral lipoatrophy [41], highlighting the relative importance of inflammation in AT and the clearance of dead adipocytes by MMe during the progression of obesity.

MMe are unique, as they are able to address specific nuances of the utilization of dead adipocytes. First, adipocytes are very large, and macrophages are unable to phagocytose them. Therefore, adipocyte clearance occurs through the formation of extracellular lysosomal compartments between many macrophages and one adipocyte, known as a crown-like structure [42]. Second, triglycerides (TGs) are the predominant intracellular components of adipocytes. During utilization, macrophages are exposed to FFAs, such as palmitate, which cause macrophage activation, further production of pro-inflammatory cytokines and inflammation [40]. Therefore, the clearance of dead adipocytes [43] causes the spread of inflammation. FFAs released by dead adipocytes stimulate the phenotypic differentiation of MMe and lead to the activation of lysosomal exocytosis, lipid metabolism and inflammation. Lysosomal exocytosis stimulates the release of FFAs while decreasing the number of TG droplets [44]. Increased expression of lipid metabolism-related genes (ABCA1, CD36 and PLIN2) helps macrophages efficiently process excess fat, and inflammation attracts other macrophages to the crown-like structure to improve adipocyte utilization. However, prolonged exposure to inflammation contributes to damage to metabolic organs (such as AT and the liver). It stimulates IR in obesity by increasing the production of pro-inflammatory cytokines, such as TNF-α [45]. Thus, MMe regulate the process of tissue inflammation in two directions by utilizing dead adipocytes and producing pro-inflammatory cytokines.

Mox have been studied mainly in the context of atherosclerosis, where the oxidation of accumulated low-density lipoprotein (LDL) leads to tissue accumulation of OxPLs, causing macrophage polarization towards a phenotype dependent on the transcription factor Nrf2 [29]. OxPLs are the main lipids stimulating the development of macrophages with the Mox phenotype [29] in individuals with various diseases, including atherosclerosis, diabetes, cancer, Alzheimer’s disease and rheumatoid arthritis, and in the pathophysiology of ageing. Macrophages recognize OxPLs through various mechanisms involving intra- and extracellular receptors. This recognition leads to the phenotypic adaptation of macrophages to a specific pathological process. Thus, Serbulea V. et al. (2018) characterized the phenotype of OxPL-induced Mox macrophages in atherosclerotic lesions from mice and observed differences from the M1 and M2 macrophages [46] and the expression of the surface markers sulfiredoxin-1 (Srnx-1) and thioredoxin reductases-1 (Txnrd-1) [47] (Figure 2). Mox differentiation depends on Nrf2 activation, which exerts anti-inflammatory effects. Nrf2 is activated by redox control either directly or via Kelch-like ECH-associated protein-1 (Keap1) [48]. Additionally, Nrf2 activates protein kinase-C (PKC) [49]. Nrf2 translocates into the nucleus and induces the expression of genes involved in the synthesis of antioxidant enzymes [50], such as heme oxygenase-1 (HO-1), glutamate–cysteine ligase modifier subunit (GCLM), glutathione S-transferase (GST) and Txnrd-1 [51] (Figure 2). All of these molecules are also specific markers for Mox. In summary, Nrf2 mediates the phenotypic polarization and controls the redox status of Mox. The transcription factor Nrf2 has been shown to induce the expression of HO-1 with anti-apoptotic and anti-inflammatory effects. HO-1 is one of the specific markers of Mox and its expression is induced by various stimuli, including lipid mediators [48]. Thus, the ability of Mox to protect against cell death is reduced due to the absence of Nrf2 or inhibition of HO-1 enzymatic activity. Based on accumulating evidence, impaired Nrf2 expression leads to various diseases associated with oxidative stress, inflammation and xenobiotic metabolism in mice [52]. Therefore, Nrf2, and hence Mox, are assumed to play a protective role in pathological processes (Figure 2).

In addition to protective functions, Mox also exhibit a pro-inflammatory phenotype. For example, the expression of pro-inflammatory genes, such as cyclooxygenase-2 (COX-2) and IL-1β, is induced in Mox, but to a much lesser extent than in M1 macrophages. Additionally, Mox show a decrease in phagocytic capacity compared to M1 or M2 macrophages. The inhibited phagocytic capacity of Mox contributes significantly to the aggravation of tissue damage and inflammation. Therefore, Mox are potentially involved in the progression and/or destabilization of pathological conditions, particularly atherosclerosis [53], obesity and diabetes.

Thus, in addition to M1 and M2, MMe and Mox are present in adipose tissue (Figure 1). These macrophages perform specific functions associated with the characteristics of inflammation in individuals with obesity (exocytosis of dead adipocytes, oxidation of phospholipids, etc.). However, MMe and Mox also aggravate pathological processes involved in inflammation. The unique biological properties of MMe and Mox suggest that these phenotypes may play important roles in the development of chronic inflammation. However, the metabolic profile and signaling mechanisms of these macrophages require additional studies.

## 5. Role of Macrophages in the Development of Endothelial Dysfunction

In clinical epidemiological studies, chronic stress was reported to be an independent risk factor for the development of atherosclerosis and the function of the intima [54]. One of the hypotheses is based on the effect of chronic stress on the accumulation of macrophages in the intima and the acceleration of damage to the mucous membrane of vascular endothelial cells [54]. Neutrophils, endothelial cells, platelets and fibroblasts are involved in the formation of a chronic inflammatory focus, accompanied by microcirculation disorders and angiogenic disorders. However, the main regulators of all cellular processes are immunocompetent cells and, predominantly, activated macrophages.

According to other researchers, chronic inflammation begins with the accumulation of critically activated macrophages in one site. According to Scalia R. (2013), the vascular endothelium of the microcirculation exhibits a substantial increase in the activity of inflammatory pathways that initiate the infiltration of macrophages into visceral AT in response to nutrient overload and long before weight gain and obvious obesity [55].

Monocytes that are attracted to the area of endothelial injury differentiate into macrophages that are capable of absorbing modified lipoproteins to cleanse the neointima [56]. Lipid metabolism by macrophages includes three stages: (1) absorption of cholesterol, (2) esterification and efflux and (3) the formation of “foam cells”. These cells accumulate in the artery wall as a characteristic longitudinal “fat band” in early atherosclerotic lesions.

Foam cells perform pro-atherogenic functions. They activate matrix-degrading enzymes that may lead to plaque rupture and the occlusion of blood vessels [56]. In this case, vascular smooth muscle cells migrate into the subintimal space of atherosclerotic lesions, which disrupts lipid transport and increases lipid absorption. As a result, the development of atherosclerotic plaques is exacerbated.

Macrophages play a key pathogenic role in endothelial dysfunction and its consequences. After infiltration into a tissue, monocytes differentiate into M1 or M2 phenotypes. High heterogeneity of macrophages is observed in individuals with atherosclerosis (Figure 1). OxPLs induce the differentiation of another population of macrophages called Mox. Additional details about this subpopulation are provided above. 

In atherosclerotic lesions from mice, macrophages with the M1 and M2 phenotypes account for 40% and 20% of the total population, respectively [57].

### 5.1. Macrophage Subpopulation M(Hb)

In addition to the M1 and M2 populations, a subpopulation of M (Hb) macrophages has been identified in areas of plaque hemorrhage in humans. The transition to the M (Hb) phenotype is stimulated by hemoglobin (Figure 2). M (Hb) macrophages are characterized by high levels of the mannose receptor (MR) (CD206) and CD163, a scavenger receptor for the hemoglobin/haptoglobin (Hb/Hp) complex (Figure 2). The Hb/Hp complex is involved in the clearance of hemoglobin from plaques after hemorrhage [58] and induces the secretion of anti-inflammatory cytokines, such as IL-10 via CD163/phosphoinositide 3-kinase and phosphatidylinositol 3-kinase (PI3K)/phospho-AKT pathways, in human macrophages in vitro and in tissue macrophages ex vivo [59,60]. M (Hb) macrophages are able to eliminate cholesterol by increasing the activity of the nuclear receptor X of the liver receptor (LXR) α (NR1H3), thereby preventing the formation of foam cells [61]. The increased LXRα activity also induces the expression of ferroportin, an iron exporter, thereby decreasing cellular iron concentrations and reducing ROS production by M (Hb) macrophages [61]. This decrease in ROS production in M (Hb) macrophages has been confirmed in atherosclerotic plaques in vivo [61,62,63].

### 5.2. Macrophage Subpopulation Mhem

During the endocytosis of the Hb/Hp complex, heme is released from erythrocytes, which stimulates the transition of macrophages to the Mhem phenotype (Figure 2). The main markers for Mhem macrophages are heme oxygenase 1 (HMOX1) and CD163 [62,63]. Mhem macrophages stimulate activating transcription factor (ATF)-1 in various ways, promoting LXRβ (NR1H2) and HMOX1 expression in humans. This process increases the expression of LXRα and ABCA1, which subsequently increase cholesterol efflux, associated with increased production of IL-10 and apolipoprotein (Apo) E [62,63,64]. Moreover, Mhem have an increased adaptation to plaque hemorrhage. Thus, the M (Hb) and Mhem phenotypes prevent foam cell formation and oxidative stress.

### 5.3. Macrophage Subpopulation M4 

Another type of macrophage, iron-loaded M4 macrophages, is predominantly detected in areas of neovascularization in atherosclerotic plaques (Figure 2). After migration through the endothelium, monocytes under the influence of CXCL4 differentiate into M4. The main markers of M4 macrophages are CD68+MR+ [62,63,65] (Figure 2). 

M4 macrophages are called CXCL4-differentiated macrophages that express the phenotypic markers metalloproteinase 7 (MMP7) and calcium-binding protein S100A8 [66]. At the same time, M4 macrophages do not express CD163 and exhibit low expression of the scavenger receptors CD36 or SR-1, which leads to a failure to induce the expression of the atheroprotective protein HMOX1 when cells are exposed to the Hb/Hp complex [62,63,67]. Thus, M4 macrophages have a pro-atherogenic profile and can be involved in complications of late atherosclerosis, such as acute coronary syndrome and arterial thrombosis. They produce the enzyme MMP12, which can be involved in the degradation of the fibrous coating of the plaque and the plaque destabilization. Furthermore, M4 macrophages express IL-6 and TNF-α, which increase inflammation. However, the fundamental role of M4 cells in atherogenesis is unknown and requires research [62,63].

Using scRNA-seq technology, a new type of macrophage, Trem2hi, has been identified, which is characterized by high expression of Trem2 (triggering receptors expressed on myeloid cells 2), Spp1 (secreted phosphoprotein1), Ctsl (cathepsin L) and CD9. The number of Trem2hi macrophages in plaques decreases with a high-fat diet [68]. Trem2 controls the expression of genes associated with energy metabolism and lipid catabolism [69].

Thus, in individuals with obesity, the structure and function of the cardiovascular system adapt to excess body weight. Metabolic disorders, such as obesity, are accompanied by endothelial cell dysfunction and decreased vascular density [70]. The modern paradigm argues that metabolic changes are associated with obesity secondary to endothelial dysfunction. The hypothesis regarding the ability of the endothelium to cause metabolic dysregulation itself must be revised and supplemented.

## 6. Role of Macrophages in the Neuroimmunological Intracellular Interactions of Adipose Tissue

Immune cells and the sympathetic nervous system (SNS) play important roles in metabolic homeostasis and pathology, particularly in obesity. The effect of macrophages on energy metabolism of adipocytes in AT is limited not only by direct effects, but also changes in neural signals in tissues.

The thermogenesis of AT plays an important role in inflammation in individuals with obesity. In brown AT, thermogenesis requires noradrenergic stimulation of the sympathetic autonomic nervous system [71,72,73]. Axons express tyrosine hydroxylase (TH) and release norepinephrine (NE), which stimulates lipolysis and the expression of thermogenic factors in brown adipocytes. In addition, sustained thermogenesis requires a tonic signal from NE [74]. As shown in the study by Nguyen K.D. et al. (2011), macrophages in brown and white adipose tissue (WAT) are potentially important sources of catecholamines that increase energy dissipation [75]. Intracellular staining for TH, Dopa decarboxylase (Ddc) and dopamine-β-hydroxylase (Dbh) revealed increased expression of all three enzymes responsible for synthesizing catecholamines in macrophages upon IL-4 stimulation. Thus, stimulation of macrophages by IL-4 increased the secretion of norepinephrine and adrenaline into the culture medium [75].

Qiu Y. et al. (2014) described the recruitment of macrophages into the subcutaneous white adipose tissue (scWAT) under cold stress via the chemokine receptor CCR2 and their roles as major integrators of thermogenic signals [76]. Mice lacking CCR2 or IL-4Rα in myeloid cells are unable to remodel scWAT into brown adipose tissue. The induction of TH, which limits the rate of catecholamine biosynthesis, provides a unifying mechanism. Through this mechanism, type 2 cytokines (IL-4, IL-5, IL-6, IL-10 and IL-13) and M2 macrophages support the browning of subcutaneous WAT. Thus, macrophages recruited to cold-stressed scWAT undergo alternative activation to express TH and produce catecholamines [52].

Most in vivo studies supporting the role of M2 macrophages as an important source of catecholamines are based on analyses of mice with a systemic deficiency of IL-4/13, Il4ra and signal transducer and activator of transcription 6 (Stat6) and mice with myeloid cell-specific deletions of Il4ra and TH. In all germline knockout models, metabolic disorders are caused by developmental processes and/or altered sympathetic regulation, since all of these genes are also expressed in the nervous system. Fischer K. et al. (2017) conducted a study to assess the role of macrophages in adult mice with a peripheral TH deletion and to further evaluate the regulatory effect of M2 macrophages on white, brown and beige adipocyte function [77]. Using a combination of in vivo and in vitro approaches, the authors showed that M2 macrophages did not synthesize sufficient levels of catecholamines. Hence, M2 macrophages do not play a direct role in adipocyte metabolism or adaptive thermogenesis. In particular, no changes in thermogenesis, energy expenditure or darkening of scWAT were observed after TH knockdown, and a significant effect of M2 macrophages on the functions of white and brown adipocytes was not observed. IL-4-mediated M2 polarization does not affect energy expenditure or thermogenesis in the inguinal WAT and brown AT of Il4ra -/- and Ucp1 -/- mice exposed to different ambient temperatures [77].

Additionally, these studies failed to detect TH expression in CX3CR1-positive mononuclear phagocytes, even after exposure to cold. Absolute levels of NE, as well as other intermediates or products of catecholamine synthesis, remained unchanged in M2 macrophages stimulated with IL-4 or in supernatants from IL-4-stimulated bone marrow macrophages. In a study by Fischer K. et al. (2017), TH was specifically removed from hematopoietic cells (including macrophages) of chimeric mice in an inducible manner. Furthermore, the absence of TH expression and NE production in macrophages from this mouse strain did not result in any impairment of energy metabolism, even upon exposure to cold. Thus, M2 macrophages do not significantly affect the metabolism of adipocytes and adaptive thermogenesis by producing catecholamines [77].

These contradictions are explained as an element of the reaction of the whole body to the effects of cold. Local catecholamine concentrations are increased in AT but the changes are not due to the direct synthesis of catecholamines by macrophages [78].

At the same time, macrophages are able to block the effects of bioactive catecholamine on white and brown AT through two mechanisms. WAT-specific macrophages have been described to inhibit sympathetic neuronal innervation and thereby impair the transmission of catecholamine signals [79]. Therefore, in their study, Yochai Wolf et al. (2017) established a role for macrophages in maintaining the innervation of brown AT. A mutation limiting the expression of the methyl-CpG binding protein 2 (MECP2) gene, which is necessary for the normal function of nerve cells, in macrophages led to a decrease in the production of the axonal protein TH in the tissue. This process caused a decrease in the sympathetic innervation of intercapsular brown AT, disrupting thermogenesis, changing body composition and subsequently leading to obesity [79]. The MECP2 protein is present at high levels in brain neurons and is associated with the maturation of the central nervous system and the formation of synaptic contacts [80].

Another mechanism involves the degradation of neurotransmitters. A distinct type of macrophage that is attached to or located near SNS axons engulfs and destroys NE. In particular, Pirzgalska R.M. et al. (2017) published a paper describing a new population of sympathetic neuron-associated macrophages (SAMs) that import and degrade NE through specific proteins that are absent in AT macrophages (Figure 1 and Figure 2).

Genes required for the function of neurons and adrenergic receptors are differentially expressed in these cells compared to other populations of macrophages. SAMs accumulate intracellular NE, despite the absence of NE biosynthetic enzymes. SNS activity increases NE levels and maintains the pro-inflammatory profile of SAMs. SAMs are imported and degraded by NE via the NE transporter (Slc6a2) and a degradation enzyme (monoamine oxidase). SAM-mediated clearance of extracellular NE contributes to obesity. Suppression of NE import by SAMs exacerbates obesity in ob/ob mice fed a HFD (leptin-deficient mice). In addition, neuron-associated macrophages pathologically accumulate in the nerves of the SNS of obese subjects in an organ-specific manner, functioning as an NE scavenger and exerting a pro-inflammatory effect [81].

Christina D. Camell et al. (2017) identified another population of neuro-associated macrophages (NAMs) that are closely associated with SN fibers and express TH (Figure 1 and Figure 2). By having direct access to the catecholamines produced by SN, NAMs regulate adipocyte access to NE [82].

The activity of the macrophage-mediated NE uptake and degradation system is enhanced in obesity (increased SAMs) [81] and GDF3-dependent overexpression of genes that control NE degradation in NAMs during ageing [82], potentially contributing to a decrease in energy metabolism (Figure 2).

The identification of SAM and NAM populations contributes to the ongoing controversy over the roles of macrophages in thermogenesis and obesity. At the same time, SAMs and NAMs have unforeseen immunological roles in NE homeostasis and therapeutic potential in individuals with obesity.

Thus, SNS nerves secrete NE into AT, which controls lipolysis, browning and thermogenesis in AT [83]. This SNS-signaling AT axis is suppressed by macrophages that degrade NE and play a role in SNS maintenance [79,81,82].

In addition to their involvement in catecholamine metabolism, IL-4-stimulated M2-like macrophages have been shown to secrete insulin-like growth factor 1 (IGF1), a hormone that was previously shown to be produced exclusively by hepatocytes [84]. Removal of the IGF1 receptor from myeloid cells decreases phagocytosis, increases the number of macrophages in AT, exacerbates obesity, decreases energy expenditure and leads to IR in HFD mice. IGF1 is involved in the nutrient perception response following stimulation with insulin, since IGF1R-deficient mice develop IR in the liver [85] and an IGF1 treatment improves the action of insulin [86,87]. Macrophages stimulated with IL-4 secrete higher levels of IGF1 and express IGF1R, suggesting an auto/paracrine effect of this classic hormone on macrophage function [60].

Cytokines play an important role in the mechanism regulating the functions of immune cells. Fatty acids present in a HFD induce pro-inflammatory signaling (NF-κB) in microglia [88], which leads to the secretion of cytokines (e.g., TNF-α) and chemokines, further inflammation in neurons and a subsequent decrease in neuronal responses (e.g., to leptin stimuli) [89].

AT macrophages produce various pro-inflammatory and anti-inflammatory cytokines that modulate sympathetic nerve activity, such as TNF-α, IL-1β, IL-6 and IL-10 [90]. When produced in peripheral tissues, cytokines enter the circulation and access the brain, enhancing sympathetic outflow by regulating the central nervous system. In the presence of various chemokines and cytokines, tissue innervation and the growth of nerve endings are limited.

α- and β-Adrenergic receptors (ARs) exhibit different binding affinities for catecholamines. NE, the main neurotransmitter of the SNS, binds to α-AR with greater affinity than to β-AR. Simultaneous expression of these receptors on immune cells (e.g., macrophages) provides these cells with a passive mechanism to determine the distance to the next source of catecholamines. The concentration in the immediate vicinity of a source of catecholamines (e.g., a sympathetic nerve or a catecholamine-producing TH-positive cell) is high enough to activate β-AR, whereas only α-AR is activated at a greater distance. In innate immune cells, such as macrophages, this stimulation directly modulates the anti-inflammatory effect (e.g., increased IL-10 production via β-AR) or pro-inflammatory activity (e.g., increased TNF production via α-AR). Therefore, the simultaneous expression of α-AR and β-AR on immune cells represents a mechanism to regulate inflammatory processes, depending on the distance to the source of catecholamines. One hypothesis is that the body uses this system to stimulate local inflammation by pushing sympathetic nerve fibers away from areas of inflammation [90].

However, in chronic inflammatory conditions arising in AT in individuals with obesity, an increased local level of inflammatory cytokines causes the repulsion (restriction of innervation through chemotaxis or obstruction of the growth of nerve endings) of sympathetic nerve fibers from the inflamed areas of AT or even nerve damage, depending on the severity of inflammation. However, evidence supporting the hypothesis that repulsion of the sympathetic nerves occurs in inflamed AT remains speculative [90].

Neuroimmunological interactions are particularly important in AT, where immune cells and the SNS play important roles in metabolic homeostasis and pathology, particularly in individuals with obesity. Several mechanisms have now been described by which macrophages directly (noradrenergic signaling) or indirectly (production of factors that alter the activity of the sympathetic nerves) modulate innervation of the AT and subsequently contribute to obesity.

## 7. The Role of Macrophages in Liver Pathologies Associated with Inflammation

Macrophages in the liver comprise approximately 85% of all macrophages in the body. Additionally, monocytes migrate into the organ through blood vessels in response to inflammatory stimuli [91]. Liver damage alters the microenvironment, affecting the phenotype and function of heterogeneous macrophage populations and their relationships with other cells. The pool of macrophage populations in the liver changes during damage. The rapid development of new methods in biology has shown that the division of macrophages into pro-inflammatory and anti-inflammatory phenotypes is outdated. Therefore, the populations of macrophages are very heterogeneous and plastic. Macrophages in the liver are mainly represented by Kupffer cells (KCs) and populations of migratory macrophages originating from monocyte-derived macrophages (MoMFs) and peritoneal macrophages of the subcapsular regions [92] (Figure 1).

KCs are resident liver cells located in the perisinusoidal space of Disse, where they interact closely with hepatic stellate cells (HSCs) and hepatocytes [93]. KCs deliver iron, which they receive from dying erythrocytes, to hepatocytes [94] and are involved in lipid metabolism [95].

In humans, these cells are identified by the presence of cluster of differentiation 68 (CD68+), CD14+, TLR4 and CX3CR1- [96] (Figure 2). However, if KCs are removed, monocytes serve as precursors to repopulate the niche [93]. In the mouse model of Clec4f-DTR (with a depleted pool of KCs), genes expressed in classical monocytes were suppressed 24 h after monocytes migrated to the liver and, in contrast, the expression of the transcription factors Nr1h3, Id3, Rxra and Spic, which characterize KCs, was induced (Figure 2). In liver pathologies (NAFLD), Kupffer cells can both protect against inflammation and cause it [97].

Under normal conditions, the maintenance of liver tolerance is mediated by KCs. Violation of tolerance changes the properties and composition of the microenvironment and promotes liver infiltration by migrating MoMFs [91].

MoMF populations have high plasticity and adaptability, which allows them to be one of the main participants in the immune response. Macrophages are susceptible. With changes in the microenvironment, the profile of the secreted molecules is modified, acquiring a specific functional phenotype. Metabolic reprogramming of macrophages from aerobic to anaerobic pathways occurs depending on the stimuli received from the microenvironment. The metabolic activity of M1 macrophages is generally based on glycolysis, while oxidative-phosphorylation reactions are more active in M2 macrophages [98].

KCs are distinguished from MoMFs by the expression of T-cell immunoglobulin and mucin domain containing 4 (Timd4) and stabilin 2 (Stab 2) [99,100]. Notably, this subpopulation of macrophages resides in the hepatic capsule, whereas only Kupffer cells were previously considered resident macrophages.

Blood-borne pathogens are removed by KCs, and liver capsular macrophages (LCMs) detect peritoneal bacteria and attract neutrophils to the capsule to reduce the pathogen load in the organ. LCMs are isolated as a distinct subpopulation of macrophages. This subpopulation is morphologically, anatomically and phenotypically different from KCs and expresses the CD207+ marker (Figure 2). A transcriptome analysis confirmed that this population is similar to the population of tissue macrophages in the intestine or skin which are derived from monocytes [92]. Notably, this subpopulation of macrophages also resides in the hepatic capsule, whereas only KCs were previously considered resident macrophages.

In a model of sterile liver injury, large peritoneal macrophages with the F4/80 hiGATA6+CD11b+ phenotype migrate to the liver within 1 h after injury [101,102]. Damage to various origins (thermal injury and NAFLD) is associated with the continuous release of damage-associated molecular patterns (DAMPs). The activation of these cells accelerates the course of inflammation and promotes rapid repair and maintenance of tissue integrity. Few studies have examined this population in the context of chronic inflammation. Given the important role of the microenvironment in determining the phenotype of macrophages, these cells may exhibit pro-inflammatory properties [103]. The data obtained indicate the heterogeneity and complexity of the phenotypes of macrophages involved in liver diseases.

In individuals with obesity, the balance of anti-inflammatory (M2) and pro-inflammatory (M1) macrophages changes. The generally accepted hypothesis linking obesity, IR and NAFLD is the development of chronic subclinical inflammation. Thus, the secretion of pro-inflammatory cytokines by macrophages blocks insulin signaling. Morgantini C. et al. (2019) disproved this hypothesis. The authors identified populations of resident and migratory liver macrophages in patients with obesity and IR and performed a transcriptomic analysis. In the background of obesity and IR, the expression of pro-inflammatory cytokines does not change. Moreover, liver macrophages produce insulin-like growth factor-binding protein 7 (IGFBP7). This protein regulates liver metabolism, inducing lipogenesis and gluconeogenesis. In addition, in obese patients with IR, the IGFBP7 isoform is expressed and exhibits a high affinity for the insulin receptor [104].

Biopsies from patients with inflammatory liver diseases are enriched in CD14+HLA-DRhiCD206+ macrophages that secrete TNF-α and granulocyte-macrophage colony-stimulating factor (GM-CSF). GM-CSF is a key growth factor required for the development of monocytes, macrophages and DCs and is a mediator of inflammation. The expression of CD206 is a marker for M2 macrophages that are involved in the mechanism regulating the immune response and tissue remodeling [105]. However, according to the results of a previous study, the accumulation of CD14+HLA-DRhiCD206+ cells is positively correlated with the degree of liver fibrosis. The authors suggest that the LPS produced by the intestinal flora activates CD14+HLA-DRhiCD206+ macrophages and blocking the production of GM-CSF inhibits the accumulation of the population to ameliorate liver fibrosis [106].

Liver macrophages express vitamin D receptor (VDR) at higher levels than other types of nonparenchymal liver cells (HSCs and epithelial cells) [107]. In a mouse model of obesity, agonist activation of VDR on liver macrophages transformed macrophages from a pro-inflammatory to an anti-inflammatory state, subsequently affecting inflammation, steatosis and IR [107].

The analysis of the scientific literature in recent years has significantly improved our understanding of the complexity, plasticity and heterogeneity of macrophage subpopulations in the liver, their secretion and their interrelations with other cells under pathological conditions. Modern methods of in situ imaging analyses of the expression of surface markers have revealed variations in the profile of macrophages in various areas of health and disease. A transcriptomic analysis of liver macrophages from patients with obesity and IR caused researchers to question the hypothesis of the development of IR in the background of inflammation. Macrophages secrete the IGFBP7 protein, which is capable of regulating liver metabolism in obese patients and patients with IR.

KCs and LCMs are the resident macrophages in the perisinusoidal space of Disse which reduce the pathogen load in the organ. Populations of migrating macrophages are the most numerous heterogeneous and plastic cells. The removal of KCs from a mouse model confirmed that monocytes migrate to the damaged organ and are reprogrammed into KCs. The accumulation of a population of macrophages with the CD14+HLA-DRhiCD206+ phenotype that secretes GM-CSF and TNFα contributes to liver fibrosis.

The migration of macrophages that differentiated from monocytes occurs through the bloodstream, and large peritoneal macrophages migrate through the mesothelium covering the liver. The presence of mature peritoneal macrophages contributes to the repair of the damaged organ. Based on the phenotypes of macrophages, the presence of liver pathology will be able to be determined and used as an approach for personalized medicine.

## 8. Pathophysiological Role of Macrophages in the Development of Aseptic Inflammatory Foci in the Skin of Individuals with Metabolic Pathologies

The formation of chronic, long-term, nonhealing lesions of the skin and mucous membranes of a noninfectious genesis is a characteristic of the development of metabolic syndrome. Normally, the function and repair of the skin are mediated by a certain set of cellular components, such as stromal cells, which include fibroblasts, endothelial cells and keratinocytes, and hematopoietic cells, including basophils, neutrophils, dendritic cells, monocytes, macrophages, lymphocytes and platelets [108,109,110].

Macrophages are one of the cellular subpopulations that trigger the process of chronic aseptic inflammation in the skin. Normally, one of the initial stages of the wound healing process is the recruitment of the neutrophilic pool of cells following the release of factors such as CXCL8, CXCL1 and CXCL2 from platelet α-granules. Neutrophils subsequently synthesize the chemokines CCL2 (MCP-1), CCL3 and CCL5, which are chemoattractants for circulating monocytes [111]. These recruited monocytes (M0) then begin to differentiate into functionally different pools of macrophages [112]. In the early stages of repair, the pro-inflammatory type of macrophages, M1, predominates. Their main function at this stage of inflammation is “cleaning” the wound focus through the phagocytosis of apoptotic cells, debris, etc. (Figure 2). In addition, M1 macrophages synthesize chemokines, such as CXCL12, which promote the transition to the anti-inflammatory phase of the repair process [9,113,114,115].

M2 macrophages are the main subpopulation of macrophages present in the remodeling phase and contribute to cell proliferation and restoration of the dermal matrix [116,117,118].

The methods used to identify monocytes/macrophages and the differentiation of monocytes recruited to skin wounds into macrophages vary. Recruited monocytes with the “classical” pro-inflammatory phenotype are identified by the expression of CD14 but not CD16 (analogue in Ly6C+/high mice), and their recruitment to the inflammatory foci is mediated by the CCR2-dependent pathway. CD14low/-CD16+ (analogue in Ly6C-/low mice) is considered to be a marker of anti-inflammatory monocytes associated with the activation of the CX3CR1 receptor. The precursors of circulating monocytes recruited to the wound originate in the bone marrow; these cells are characterized by the following phenotypes: CCR2high/CX3CR1low receptors, “classic type”, and CCR2low/CX3CR1high, “alternative type” [33,34,114,119].

The characteristics of differentiating macrophages that are functioning in wounds are more complex and ambiguous. Thus, resident dermal macrophages originating from progenitor cells of the yolk sac and fetal liver are identified by several surface markers, such as F4/80hi, CD64+, MERTK+ and CCR2-/low [120,121].

The pro-inflammatory pool of macrophages is identified by the F4/80+CD11c+CD206- phenotype and the expression of inducible NO synthase (iNOS/NOS2) and the CCR2 receptor, and a phenotype characteristic of macrophages of the M2 type is F4/80+CD11c–CD206+ and CD163 expression and, to a lesser extent, the expression of Arg1, due to its expression in macrophages of both phenotypes [114,119,122].

Pools of monocytes and macrophages present in the wound bed are transient, have dual phenotypes (e.g., expressing markers of pro- and anti-inflammatory phenotypes: TNF-**α**hi, IL-12hi, CCR2hi, Ly6Chi, Dectin-1med, IL-4R**α**med and CD204med) or are present as phenotypic pools that do not fit into the simplified M1/M2 scheme of classically and alternatively differentiated macrophage phenotypes, such as Mox, M4 and Mhem (Figure 1). However, their roles in targeting and promoting the terminal resolution of the process of chronic wounds currently requires additional study [116,121,123].

Metabolic disorders accompanied by a state of hyperglycemia lead to dysfunctional changes that disrupt the transition from the inflammatory phase to the remodeling phase (anti-inflammatory stage) during the healing of chronic skin and soft tissue wounds. One explanation for these disorders is the quantitative and functional changes in the cellular composition of a chronic wound. In particular, the normal function of platelets changes, resulting in changes in the number and imbalance of neutrophil functions. This change entails a deviation of the processes involved in the phased differentiation of macrophages in chronic wounds. Furthermore, if a large number of pro-inflammatory M1 macrophages persist in the wound, a long-term macrophage-mediated inflammatory response occurs [116,123,124,125,126].

One of the explanations for the chronicity of wounds in individuals with obesity and diabetes mellitus is the dysregulation of the recruitment and persistence and activation of monocytes/macrophages, as well as impaired efferocytosis processes. In the early stages of the repair of diabetic wounds, macrophage infiltration is delayed due to a decrease in CCL2 expression [126,127,128]. However, in the late stages of chronic wound healing, a large number of neutrophils and monocytes/macrophages persist in the injured site, along with an increase in the infiltration of MoMFs with the “classic” Ly6CHi phenotype during this period, which is not typical under normal conditions [128,129,130]. This effect is associated with high levels of gene expression and subsequent secretion of the key pro-inflammatory cytokines IL-1β and TNF-α and the chemokines MIP-2 and CCL2 during the late stages of the wound healing process [126,129].

An increase in the functional load on IL-1β and TNF-α leads to a decrease in the expression of one of the isoforms of peroxisome proliferator-activated receptors, PPARγ, which is associated with suppression of the efferocytosis function in M1 macrophages in chronic diabetic wounds. In knockout mouse models with impaired activity of the PPARγ receptor, a decrease in the phagocytic activity of macrophages and increased activity of the NLRP-3 inflammasome were observed in the early stages of repair, which ultimately contributed to the chronicity of wounds [124,131,132,133].

Thus, the development of metabolic syndrome is characterized by the formation of chronic, long-term, nonhealing lesions of the skin and mucous membranes of a noninfectious origin. Pools of monocytes and macrophages of the wound bed are transient, have a dual phenotype (expressing markers of pro- and anti-inflammatory phenotypes: TNF-**α**hi, IL-12hi, CCR2hi, Ly6Chi, Dectin-1med, IL-4R**α**med and CD204med) or are present as phenotypic pools that do not fit into the simplified M1/M2 scheme of classical and alternative differentiation, such as Mox, M4 and Mhem, but their roles in promoting and regulating the terminal resolution of the healing process of chronic wounds requires additional study.

## 9. Conclusions

Macrophages are involved in the development of various metabolic pathologies associated with aseptic inflammation. In each tissue, these cells play a main role in the development of obesity and its complications, namely T2DM, atherosclerosis, microvascular lesions, liver diseases (NAFLD), neurohumoral disorders and lesions of the skin and mucous membranes leading to the formation of ulcers.

Macrophages are involved in the maintenance of homeostasis and the regulation of inflammatory and regenerative processes. Monocytes have been shown to transition from one activated state to another to differentiate into macrophages, and then their differentiation pathways diverge to M1- and M2-like types of mature macrophages [33,34]. However, the plasticity of monocytes has also been observed, as these cells first polarize into anti-inflammatory monocytes and then terminally differentiate into pro-inflammatory macrophages [33,35].

Usually, two phenotypes of macrophages are distinguished: M1 and M2. However, other types have been identified in AT that regulate the inflammation of AT in individuals with obesity. MMe macrophages play one of the key roles in the pathogenesis of obesity and IR due to their ability to utilize dead adipocytes. MMe both aggravate and reduce the process of inflammation in the tissue.

The unique biological properties of Mox, the development of which is mediated by OxPLs, suggest that cells with this phenotype may play an important role in the development of chronic inflammation. However, the metabolic profile of Mox has not been investigated. Additionally, the mechanisms by which OxPLs activate inflammatory signaling pathways and metabolic changes in Mox remain poorly understood.

Macrophages play a key pathogenic role in the development of endothelial dysfunction and its consequences. The migration of immune cells has been suggested to contribute to the formation of vascular pathologies. However, other researchers have shown that the root cause of vascular disorders is the migration of macrophages into the vascular endothelium and the activation of inflammatory pathways long before weight gain and obvious obesity.

In addition to M1 and M2 macrophages, a subpopulation of M (Hb) macrophages has been identified in areas of hemorrhage in atherosclerotic plaques in humans. The Hb/Hp complex is involved in the clearance of hemoglobin from plaques after hemorrhage and induces the secretion of anti-inflammatory cytokines. M (Hb) macrophages eliminate cholesterol, reduce ROS levels and prevent the development of foam cells. Macrophages with the Mhem phenotype increase cholesterol efflux and prevent foam cell formation and oxidative stress. Another type of macrophage, iron-loaded M4 macrophages, predominates in areas of neovascularization in atherosclerotic plaques and has a proatherogenic profile. The migration of monocytes and macrophages into inflammatory foci and their sequential production of pro- and anti-inflammatory cytokines and factors promotes angiogenesis and the resolution of inflammation. If the damaging factor persists (for example, hyperglycemia), then chronic inflammation associated with an angiogenic reaction develops to provide blood to the inflamed tissue.

Immune cells and the SNS play important roles in metabolic homeostasis and pathology, particularly in individuals with obesity. The effect of macrophages present in the AT on energy metabolism in adipocytes is limited not only by direct effects but also changes in neural signals in tissues. Neuroimmunological interactions are particularly important in AT, where immune cells and the SNS play important roles in metabolic homeostasis and pathology, particularly in individuals with obesity. The SNS releases NE into AT, which controls lipolysis, browning and thermogenesis in AT [83]. This SNS-signaling adipose tissue axis is suppressed by macrophages which degrade NE and play a role in SNS maintenance. Several mechanisms by which macrophages directly (noradrenergic signaling) or indirectly (production of factors that alter the activity of the sympathetic nerves) modulate innervation of the AT and subsequently contribute to obesity have now been described.

The liver contains approximately 85% of all macrophages in the body [91]. In addition, monocytes are able to migrate through blood vessels to the organ in response to inflammatory stimuli. Liver damage alters the microenvironment, affecting the phenotype and function of heterogeneous macrophage populations and their relationships with other cells. Macrophages in the liver are mainly represented by KCs and populations of MoMFs and subcapsular peritoneal macrophages. The violation of tolerance changes the properties and composition of the microenvironment and promotes hepatic infiltration by migrating MoMFs. LCMs detect peritoneal bacteria and attract neutrophils to the capsule to reduce the pathogen load in the organ. Inflammation has been described as the root cause of the development of NAFLD. However, other authors have found that macrophages themselves regulate their metabolic status in the liver. Thus, a transcriptomic analysis of liver macrophages from patients with obesity and IR caused researchers to question the hypothesis of the formation of IR in the background of inflammation.

The formation of chronic, long-term, nonhealing lesions of the skin and mucous membranes of noninfectious genesis is a characteristic of the development of metabolic syndrome. Pools of monocytes and macrophages of the wound bed are transitional, have a dual phenotype (e.g., expressing markers of pro- and anti-inflammatory phenotypes: TNF-**α**hi, IL-12hi, CCR2hi, Ly6Chi, Dectin-1med, IL-4R**α**med and CD204med) or are present as phenotypic pools that do not fit into the simplified M1/M2 scheme of classical and alternative differentiation, such as Mox, M4, and Mhem.

Recent studies have described phenotypes of macrophages that do not fit into the simplified M1/M2 scheme of classical and alternative differentiation, such as MMe, Mox, M4 and Mhem. The primary cause of some metabolic pathologies, such as NAFLD and disorders of the endothelium of microvascular circulation, has been precisely identified as the recruitment of macrophages with an altered phenotype, which is probably typical for many other metabolic pathologies in individuals with obesity. Macrophages are ubiquitous, making them the best candidates for cell therapy. Since the entire spectrum of phenotypes and functional activity of macrophages in tissues is represented by essentially one type of cell, these cells provide opportunities to develop multidirectional therapeutic strategies for the treatment of obesity-associated diseases. 

Drugs targeting macrophages must be used with caution. Using modern technologies, populations of macrophages with a protective effect on inflammatory processes will be developed. In situ reprogramming of macrophages may be a significant focus in the future to reduce mortality.
